# Recruitment strategies and yields for the Pathobiology of Prediabetes in a Biracial Cohort: a prospective natural history study of incident dysglycemia

**DOI:** 10.1186/1471-2288-13-64

**Published:** 2013-05-10

**Authors:** Sotonte Ebenibo, Chimaroke Edeoga, Ann Ammons, Nonso Egbuonu, Samuel Dagogo-Jack

**Affiliations:** 1 Department of Medicine, Division of Endocrinology, Diabetes and Metabolism, University of Tennessee Health Science Center, Memphis, TN, USA; 2 University of Tennessee Health Science Center, 920 Madison Avenue, Suite 300A, Memphis, TN 38163, USA

**Keywords:** African American, Advertising, Community outreach, Racial differences, Longitudinal retention

## Abstract

**Background:**

The Pathobiology of Prediabetes in A Biracial Cohort study is a prospective evaluation of the transition from normal to impaired glucose regulation among African American and Caucasian adults with parental type 2 diabetes. This report describes recruitment strategies and relative yields for the 376 enrolled subjects.

**Methods:**

Recruitment occurred over 3.4 years, with clinical and metabolic assessments during 2.1-5.5 years of quarterly follow-up. The major recruitment sources were advertisements, community outreach, and clinical facilities. Advertisements included newspaper, television, radio, Internet, distributed brochures, utility bill inserts, and direct mailing. Community outreach included screening events during religious gatherings and health fairs, and referral by friends and families. The category of clinical facilities covered all subjects referred by health workers or recruited through area clinics and hospitals.

**Results:**

57.7% of participants were African American and 42.3% were Caucasian; the mean age (± SD) was 44.2 ± 10.6 years, and ~70% were female. Advertisements yielded 52.4% of all participants, compared to 34.8% from community outreach and 12.8% from clinical facilities (P for trend < 0.0001). More Caucasians than African Americans cited advertising as the source of study information, whereas more African Americans than Caucasians cited community outreach. The accrual from clinical facilities was similar in both groups.

**Conclusions:**

Advertisements and community outreach were robust recruitment sources for assembling a diverse longitudinal diabetes offspring cohort, but each had differential yields in African Americans and Caucasians. Thus, a multifaceted approach comprising passive and active components is needed to recruit a multiracial clinical research population.

## Background

The Pathobiology of Prediabetes in A Biracial Cohort (POP-ABC) study is a prospective evaluation of the natural history of impaired glucose regulation in African Americans and Caucasians. National surveys indicate a higher prevalence of type 2 diabetes in African Americans than Caucasians [1,2]. Prediabetes, defined as impaired fasting glucose (IFG) and/or impaired glucose tolerance (IGT) [3], is an intermediate stage during the development of T2DM. Persons with prediabetes progress to type 2 diabetes at an annual rate of ~10%, which is similar for African Americans, Hispanics, Asian Americans, Native Americans and Caucasians [4]. The lack of ethnic disparities in incident type 2 diabetes among persons with prediabetes [4] was surprising, given the marked ethnic disparities in the prevalence of type 2 diabetes. The POP-ABC study was initiated in response to a National Institutes of Health request for studies to determine what metabolic or physiologic processes account for the pathogenetic pathways leading to ethnic and racial disparities in type 2 diabetes.

The objective of the POP-ABC study is to determine whether ethnic disparities occur more proximally, during the transition from normoglycemia to prediabetes. All participants are adult offspring of parents with type 2 diabetes, which allows for the detection of ethnic and environmental factors among persons at comparable hereditary risk for diabetes. Eligibility criteria include age 18–65 years, history of type 2 diabetes in one or both biological parents, normal fasting plasma glucose (FPG) and/or normal glucose tolerance (NGT), and African American or Caucasian status. We documented parental history of type 2 diabetes using a standardized diabetes-focused history, including information on the number of affected biological parents, parent’s gender, age at diagnosis, use of diabetes medications, diabetic complications, and contact information of the parents’ diabetes care provider. Enrollment in POP-ABC began in September 2006 and ended in February 2010. Participants underwent assessments (including measures of dietary and exercise behavior, clinical examination, glucose tolerance, insulin sensitivity, beta-cell function, body composition, energy expenditure) during 2.1-5.5 years of quarterly follow-up until March 2012. The primary outcome is the occurrence of prediabetes (impaired fasting glucose {IFG} and/or impaired glucose tolerance {IGT}), as defined by the American Diabetes Association criteria [3]. The design and methods of the study have been published [5].

The presence of prediabetes or diabetes may be a motivation to participate in long-term intervention trials for prevention of diabetes or optimization of glycemic control [6-10]. On the other hand, longitudinal studies of initially healthy cohorts have tremendous value in unraveling the epidemiology and natural history of disease and identifying targets for the development of preventive interventions [11].

Healthy subjects generally participate in clinical research studies as “controls” for measurements obtained from patients suffering from a specific disorder, or as the target study population for observational or interventional protocols. Regardless of the exact design of the study, it is desirable for healthy persons to be encouraged to participate in clinical research for a number of reasons. First, measurements or responses obtained from healthy subjects provide normative data with which to contrast results obtained from patients affected by the disease or condition being studied. For example, the documentation of the presence and severity of insulin resistance in patients with type 2 diabetes is made possible by knowledge of the expected (“normal”) insulin-mediated glucose disposal among healthy control subjects. Second, in observational studies, such as the Framingham Study (11), repeated measurements in a cohort of initially healthy subjects over several decades have helped define the risk factors and natural history of heart disease, diabetes, and other cardiometabolic disorders. Thirdly, knowledge of the natural history of a given disease process has been of critical value in the development of effective strategies for screening, prevention, early detection and treatment. Fourth, depending on the specific study, participants in clinical research receive several benefits, including free physical examination, stipends, laboratory tests, medications and other supplies. Thus, although time consuming and often intrusive, participation in clinical research by healthy persons does have benefits at the societal and personal levels that often outweigh the risks and costs to the individual.

However, there is limited information on strategies for recruiting healthy subjects into long-term studies that do not offer health interventions or direct health benefits. Indeed, the recruitment and retention of healthy individuals in observational studies that entail frequent visits for extensive, repeated metabolic assessments can prove challenging. Successful approaches to overcoming such challenges, especially among individuals from underserved minority populations, would be most desirable. In this report, we describe the recruitment methods, strategies, and relative yields for the 376 healthy African Americans and Caucasians enrolled in the POP-ABC study.

## Methods

The POP-ABC study utilized recruitment strategies that have previously been reported to be effective in clinical studies [12-14]. These strategies included direct mail, advertisements, and screenings at health fairs, religious gatherings and other community settings. The principal strategies employed in the POP-ABC study are described below, and the relative yields from the different recruitment sources are presented in the Results section of this paper.

### Organization of Recruitment

A recruitment coordinator was given primary responsibilities for identifying and developing recruitment opportunities, implementing recruitment strategies, and leading the study group at such events, under the overall direction of the principal investigator (PI) (SD-J). Recruitment brochures and flyers with pertinent information were developed, and ~200,000 copies were distributed through various channels. All study personnel had hand-on experience with one or more of the recruitment initiatives, and participated in community events. Recruitment progress and ideas for new initiatives were discussed during weekly research group meetings. The POP-ABC study protocol was approved by the University of Tennessee Health Science Center (UTHSC) Institutional Review Board. All subjects gave written informed consent prior to the initiation of the study, which was conducted in accordance with the principles of the Declaration of Helsinki [15].

### Direct mail and distributed printed material

At the inception of the study, recruitment brochures were mailed directly to 5,000 persons selected from an internal database of individuals who had expressed interest in participating in clinical research. Also, 20,000 brochures and postcards targeting potential participants were mailed to Memphis-area households in selected zip codes. A second wave of direct mail (20,000 pieces) was sent out to a different set of households approximately 4 months later. In addition, flyers and brochures were distributed at strategic locations on the UTHSC campus and also sent to area health facilities, physicians with high-volume diabetes practices, pharmacies, educational institutions, local libraries, grocery stores and other community outlets. At UTHSC-affiliated clinics, brochures were distributed to patients with diabetes who might have eligible offspring. In all, ~200,000 flyers and brochures were distributed during the recruitment period spanning September 2006 – February 2010. As enrollment progressed, each participant was given 10 brochures at the end of each study visit for distribution to relatives and friends.

### Advertisements and media exposure

Paid advertisements were placed in the dominant Memphis-area newspaper (*The Commercial Appeal*) on four occasions during the initial 2.5 years of recruitment. On each occasion, the advertisement ran on three non-consecutive days each week. Paid advertisements also were placed in smaller newspapers targeting residents of the suburbs. We also advertised in monthly utility bill inserts distributed to households during August 2007 – December 2007, and throughout 2008 and 2009. A total of 25 advertising spots were purchased in local television stations during the recruitment period. The study was also advertised on the Internet and via the campus-wide UTHSC email listserve to all university employees. In addition to the paid advertisements, mass media exposure was achieved at no cost to the study through interviews given by the PI on four local television and two local radio stations, as well as numerous articles, interviews, and news features carried in local newspapers. The *Commercial Appeal* published a front page story and an editorial on prediabetes in which the PI was extensively quoted. Gratuitous media coverage of the study also appeared in numerous publications, including the *Memphis Business Journal, Memphis Medical News, Memphis Health & Fitness, Memphis Silver Star News, Daily Helmsman, Germantown & Collierville Appeal, Commercial Appeal.com, The Democrat, Physicians Practice Magazine, Tri-State Defender, Collierville Herald, and UTHSC News.* We were also featured in the *Mid-South Wellness Guide, Good Health, American Diabetes Association Newsletter, Diabetes Forecast, Body + Soul, Upscale, and JET.* The latter has a large African American readership.

### Community outreach and screening events

After 6 months of recruitment experience, the accrual from mass mailing, advertising, and media exposure fell short of projected enrollment targets. It thus became necessary to initiate direct action in the community. A letter describing the purpose of the POP-ABC study was mailed to area churches, businesses, and colleges. Included in the letter was an offer by the PI and co-investigators to deliver onsite diabetes awareness presentations, and provide additional information regarding the study. There was an excellent response to these solicitations, and beginning in April 2007 until the end of the recruitment (February 2010), we participated in 72 unique community outreach events. These included presentations and screenings at 30 area churches, 23 community health fairs, 9 work place events, and a total of 10 corporate retreats and other civic gatherings.

### Community screening procedures

At a typical community outreach event, the PI, recruitment coordinator, or a study group member would make a lay presentation on the subject of diabetes and pre-diabetes and provide background on the POP-ABC study, followed by a question-and-answer session. Thereafter, members of the audience interested were invited to visit booths or tables manned by study personnel at various break-out stations. Specially designed banners emblazoned with the study logo (picture of a hawk, with the phrase *“Watching Your Health Like a Hawk”* and contact telephone numbers) were displayed on the booths and in front of tables during break-out sessions. The logo was also affixed to T-shirts and embroidered on baseball hats, which were handed out as promotional items. Interested persons were provided information regarding the study, and asked to complete a brief pre-screening questionnaire focusing on age, race/ethnicity, personal and parental history of diabetes, medication history, and their interest in undergoing blood glucose screening. Potentially eligible subjects who expressed interest in undergoing blood glucose screening received a copy of the informed consent form to study at home. Those who remained interested after reading the informed consent document contacted the study group to schedule their initial visit to the UTHSC General Clinical Research Center (GCRC).

Other potential research participants called the study telephone number provided in flyers, brochures, newspapers, television and radio broadcasts, and other advertisements. A telephone pre-screening questionnaire similar to the one completed by persons encountered during community events was administered, and the informed consent form was mailed to persons who appeared to be potentially eligible. Those who remained interested in participating, after reading the informed consent, contacted the study group to schedule their first visit to the GCRC. After finalizing the informed consent procedure in person, an initial visit protocol, including a standard 75-g oral glucose tolerance test (OGTT), was performed. Persons who met the glycemic and other eligibility criteria, as previously described [5], were enrolled. The source of information about the study was documented for each participant.

### Statistical analysis

Data are presented as mean ± SD. Chi-square tests were performed to assess differences by gender and race/ethnicity in the yields from the various recruitment sources, and Mantel-Haenszel chi-square tests were used to analyze the trends across three defined age groups [16]. The analyses were performed using SAS Software, version 9.2 (SAS Institute, Cary, NC). P < 0.05 was accepted as significant.

## Results

**Figure 1:**
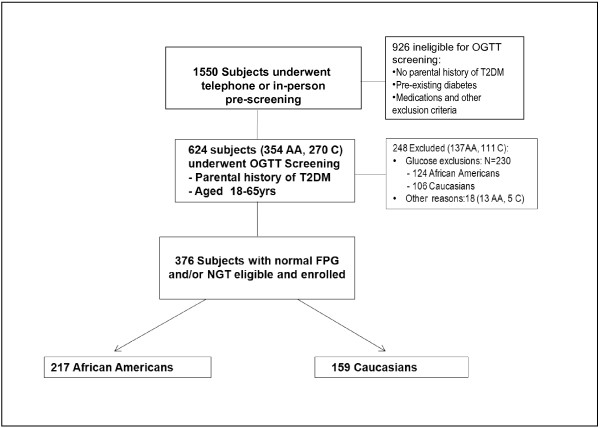
**Screening and enrollment data for the POP-ABC study.** AA, African American; C, Caucasian; OGTT, oral glucose tolerance test; T2DM, type 2 diabetes mellitus.

Pre-screening questionnaires were administered to 1550 individuals via the telephone or in person during community events. A total of 926 persons were not invited for OGTT screening because they met exclusion criteria. The major reasons for exclusion at the level of pre-screening were lack of parental history of type 2 diabetes, current diagnosis of diabetes or use of antidiabetes medications, race/ethnicity status other than African American or Caucasian, and planned relocation from the Memphis area within 5 years [5]. A total of 624 persons without a history of diabetes but whose biological parents had type 2 diabetes underwent OGTTs. Of those, 376 subjects who met all eligibility criteria were enrolled in the study. Of all participants, 217 (57.7%) were African American and 159 (42.3%) were Caucasian; their mean (± SD) age was 44.2 ± 10.6 years, and the cohort was ~70% female. Figure 1 summarizes the pathway to final enrollment.

### Recruitment progress over time

A total of 27 participants were enrolled during the first 3 months of the study, and 120 subjects were enrolled in the first year. Thereafter, an average of 12 participants were enrolled monthly (range 2–23 subjects) throughout the recruitment period, except during November – December 2009 when only one subject was enrolled per month. Active advertising and community recruitment efforts ceased in September 2009, but eight subjects who missed appointments in late 2009 were enrolled during January-February 2010.

### Sources of recruitment

A pre-screening questionnaire was completed for every potential participant who made contact with the study team. The answer to the question “How did you hear about the study?” was recorded as the primary source of information for each potential participant. The results from the 376 enrolled participants are presented in this report. The yields of participants from the various primary sources of information were as follows: direct mail 5.9%; print (newspapers, magazines, utility bill inserts and advertisements placed in grocery stores and other outlets) 30.6%; community outreach events 24.5%; radio, television, and Internet 9.8%; referral by friends and relatives 10.4%; clinical facilities 12.8%; and flyers/brochures 6.1% (Table 1).

**Table 1:** Percentage yields from recruitment sources in the POP-ABC study and the diabetes prevention program

**POP-ABC study**				**Diabetes prevention program**	
	**All**	**Caucasian**	**Afr. American**	**Caucasian**	**Afr. American**
Direct Mail	5.9	10.7	2.3	29.6	39.8
Print	30.6	35.8	26.7	18.2	9.0
Screening event	24.5	12.6	33.2	8.2	13.7
Radio/TV/Internet	9.8	8.8	10.6	9.4	10.0
Relative/Friend	10.4	11.3	9.7	6.5	7.8
Clinical referral	12.8	12.6	12.9	6.4	7.4
Flyer/Brochure	6.1	8.2	4.6	3.3	0.9

In the Diabetes Prevention Program (DPP) ^13^ phone solicitations (a method that was not used in the POP-ABC study) yielded 0.9% of Caucasian and 1.3% of African American recruitment. The Internet was not a recruitment source for the DPP. The DPP columns do not add up to 100% because of missing values and “other” category. The POP-ABC category of Flyer/Brochure is equivalent to the DPP category of Poster/Newsletter^13^. POP-ABC = *Pathobiology of Prediabetes in A Biracial Cohort.*

### Comparison of POP-ABC and DPP recruitment data

The POP-ABC study and the Diabetes Prevention Program (DPP) [4,17] both targeted demographically diverse populations of nondiabetic subjects for enrollment into a longitudinal study. Table 1 compares African Americans and Caucasians in the POP-ABC and DPP studies with regard to their primary sources of information. The top three sources of recruitment information for the DPP were direct mail, print advertising, and screening events [17], compared to print advertising, screening events, and clinical facilities for the POP-ABC. Direct mail, the leading source of DPP enrollees, was markedly less effective in the POP-ABC study. In both the DPP and POP-ABC study, more Caucasians than African Americans reported print media as their primary source of information. Similarly, in both studies, African Americans were at least twice as likely to cite community outreach as their source of study information compared to Caucasians. Approximately 10% or less of participants cited radio or television advertisements or relatives/friends as the source of study information in both the DPP and POP-ABC studies. Distributed printed materials (posters, newsletters, flyers, brochures) were the least cited source of information for participants in both studies (Table 1).

### Major categories of recruitment sources

**Figure 2:**
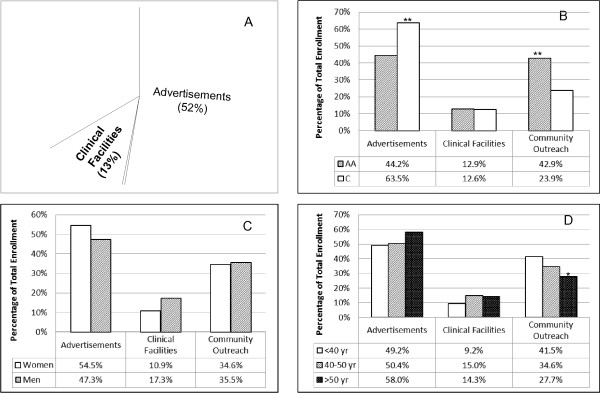
**Distribution of enrolled participants by major recruitment sources (A) and by race (B), gender (C) and age (D).** The yields from the different sources were significantly different from a hypothetical distribution of 1:1:1 (chi square P < 0.0001). Advertising yielded more participants than community outreach (P = 0.0003) or clinical facilities (P < 0.0001). Community outreach was significantly more productive than accrual from clinical facilities (P < 0.0001). * P < 0.05, ** P < 0.001.

The primary sources of information about the study elicited from POP-ABC participants were classified into three major categories: Advertisements, Community Outreach, and Clinical Facilities. The category of advertisements included television, newspaper, radio, the Internet, distributed brochures and flyers, utility bill inserts, direct mailing, and displays at grocery stores, pharmacies and other outlets. Community outreach involved direct contacts during religious gatherings, health fairs, educational presentations, other encounters, and referral by friends and families. The category of clinical facilities covered all subjects referred by health workers or recruited through area clinics and hospitals. Figure 2 shows the distribution of enrollees across the three major categories of recruitment sources. For the whole study cohort, advertisements were the most frequently cited source of information by participants, accounting for 52.4% of enrollment. Within the advertisement category, newspaper (13%) and utility bill inserts (16.5%) were more frequently cited than the Internet (2.4%) or radio (0.5%). Community outreach was cited as the source of information by 34.8% of participants and clinical facilities were cited by 12.8% of enrolled subjects. The yields from the three major categories of sources were significantly different from a hypothetical distribution of 1:1:1 (chi square P < 0.0001). Advertising yielded more participants than did community outreach (P = 0.0003) or clinical facilities (P < 0.0001). Community outreach was significantly more productive than accrual from clinical facilities (P < 0.0001).

### Recruitment sources by race, gender and age

The distributions of the accrual from the three major categories of recruitment sources by race, gender and age groups are shown in Figure 2. More Caucasians than African Americans (63.5% vs. 44.2%, P <0.001) cited advertising as the source of information about the study, whereas more African Americans than Caucasians (42.9% vs. 23.9%, P < 0.001) cited community outreach. The low yield from clinical facilities (~13%) showed no ethnic disparities. The distribution of participants in the major categories of recruitment sources was similar in men and women (Figure 2). There were no age-group differences among participants who cited advertisement or clinical facilities as their sources of information about the POP-ABC study. However, community outreach was more frequently cited as the source of information by participants younger than 40 years of age compared to older participants (Mantel-Haenszel chi-Square P = 0.0225 for linear trend) (Figure 2).

### Costs of recruitment

The POP-ABC was funded by a grant from the National Institutes of Health that covered personnel salaries and other costs. A full-time position was assigned to coordinate recruitment efforts during the initial ~3.5 years of the study. The entire study team (including the PI and associates) was involved in the community recruitment events. Most community events were manned by at least three study personnel. However, there was no extra cost to the project, as these were salaried positions paid directly from the study grant. A flyer summarizing the study goals, eligibility criteria, and contact telephone numbers was the main instrument of dissemination of recruitment information. When tri-folded, the flyer becomes a brochure that can be mailed. To save costs, desktop publishing was used to produce flyers in-house and a tri-fold machine was purchased to enable conversion of flyers to brochures.

The approximate cost of all flyers and brochures used during the recruitment exercise was $28,000. The cost of three waves of mass mailing was $16,700, not including personnel time in the preparation of address labels and related tasks. The total cost of paid advertising was $28, 600. Of these, newspapers (11 episodes amounting to $8,800), utility bill inserts (27 episodes amounting to $11, 550), and television (25 episodes costing $6250) were the major expenditures. Recruitment through clinical facilities involved delivery of study flyers and brochures to area physicians, clinics and hospitals. These duties were accommodated during regular working hours at minimal additional cost to the project. Adding the cost of 3.5 years of hiring a recruitment coordinator (~$200,000) to the costs of production of flyers/brochures, paid advertisements, and mass mailing, brings the estimated recruitment expense to ~ $273,300. An average of three research personnel attended 72 community outreach events and devoted ~ 1296 hours to direct recruitment efforts at an estimated cost of $22,836, bringing the total recruitment costs to $296,136 or $788 per subject enrolled. The monetary value of the free media coverage was discounted.

## Discussion

The objective of the POP-ABC Study is to determine the natural history of prediabetes among initially normoglycemic persons, and to assess the role of race/ethnicity. To our knowledge, this is the only prospective study of initially normoglycemic African American and Caucasian offspring of parents with type 2 diabetes. The identification of such individuals required population-based screening and recruitment strategies. Besides the potential barriers to recruiting healthy subjects into a long-term observational study, additional barriers for minority and low income or low literacy populations include transportation, meals, child care, time off-work, unfamiliarity with the research environment, and historical distrust of medical research [18-21]. The provision of clear written directions to the GCRC (with maps), flexible scheduling, free parking, and snacks/meals during study visits served to address some of these barriers.

Of the three major categories of recruitment sources, advertisements and community outreach were much more effective in driving enrollment compared with clinical facilities. We had hoped that focusing on older patients at diabetes clinics would have served as a conduit to recruiting their healthy offspring. Our experience, and that of the DPP [17], shows that clinical referral is a low-yield source of healthy research subjects, perhaps because such persons are underrepresented among routine recipients of outpatient and inpatient services. By contrast, clinical centers are a rich recruitment source for studies that target patients with specific diseases. Our goal was to enroll a population-based sample comprising 50% non-Hispanic whites and 50% non-Hispanic blacks. Remarkably, the African American target was reached and exceeded with relative ease. The enrolled cohort comprised 57.7% African American and 42.3% Caucasian. Several factors could have contributed to our uncommon success in enrolling African Americans- high concentration of African Americans in the Memphis area, ethnic diversity among the investigators, aggressive use of community outreach, and social marketing [21,22].

Similar to the DPP [17], our study sought individuals without diabetes for enrollment into a diabetes-related long-term follow-up study. However, POP-ABC included only normoglycemic subjects who had biological parents with type 2 diabetes, whereas the DPP enrolled prediabetic persons with or without a family history of diabetes. Moreover, the DPP offered diabetes prevention interventions, whereas POP-ABC is an observational study. Nonetheless, a comparison of the recruitment data from both studies provides insight into the successful assembly of multiethnic cohorts. Print advertising and community events were the top two recruitment sources for the POP-ABC study, and the second and third leading sources for the DPP. Notably, in both studies, community outreach attracted more African Americans than Caucasians, whereas print advertising attracted more Caucasians than African Americans. Radio, television and the Internet were less productive recruitment sources for the POP-ABC and DPP studies (although the Internet was not utilized in the latter) (Table 1). Interestingly, direct mail was cited as source of information by ~30% of Caucasians and ~40% of African Americans in the DPP, whereas only 11% of Caucasians and 2% of African Americans in the POP-ABC study cited that source. The reason for the large difference is unclear, but the DPP contracted with a national public relations firm that orchestrated a centralized publicity program, whereas no such facility was available to the POP-ABC study.

Our experience and that of the DPP indicate that community outreach is a robust source of research subjects for a healthy cohort study. However, implementing community outreach programs is more demanding than mass mailing or media advertising. In addition to the services of a full-time recruitment coordinator, recruiting via community outreach required ~1300 hours of POP-ABC study personnel time. Our estimated recruitment cost of US $788 per subject was somewhat lower than the US$1075 reported for the DPP [17]. However, both figures likely underestimate the true cost of finding and enrolling healthy or asymptomatic research subjects. For smaller studies of short duration, an initial recruitment program based on passive advertising seems reasonable. However, for large prospective studies, particularly those targeting a diverse population, a combination of community outreach and advertising would be advisable.

## Conclusion

In conclusion, using a multifaceted approach, we have recruited and retained a large cohort of African Americans and Caucasians with parental diabetes. This unique cohort of persons at comparable genetic diabetes risk should improve the detection of environmental factors that interact with race and ethnicity in the pathogenesis of early dysglycemia. The specification of incident prediabetes, rather than diabetes, as the primary endpoint enables the identification of individuals who may benefit from timely interventions. Thus, the POP-ABC study will generate new knowledge on the rates and predictors of the transition from normal to impaired glucose regulation in African Americans and Caucasians at high risk for type 2 diabetes. By design, participation in the POP-ABC study was limited to African Americans and Caucasians with parental type 2 diabetes, which is a limitation regarding generalizability of the study’s findings to other demographic groups. Nonetheless, a fuller understanding of the pathobiology of prediabetes should facilitate proper targeting of interventions to prevent diabetes [4,8-10], or reverse prediabetes and restore normal glucose regulation [23,24] in high-risk groups.

## Competing interests

The authors declare that they have no competing interests.

## Authors’ contributions

SD-J developed study concept and design, supervised study execution, and wrote manuscript. AA collected data and reviewed and revised manuscript. SE performed statistical analysis, reviewed and revised manuscript. CE collected data and reviewed and revised manuscript. NE collected data and reviewed and revised manuscript. All authors read and approved the final manuscript.

## Authors’ information

SE, CE and NE are Research Associates, AA is a Research Assistant, and SD-J is Professor of Medicine and Director, all in the Division of Endocrinology, Diabetes and Metabolism, University of Tennessee Health Science Center, Memphis, TN.

## Pre-publication history

The pre-publication history for this paper can be accessed here:

http://www.biomedcentral.com/1471-2288/13/64/prepub

## POP-ABC Research Group

Current: Samuel Dagogo-Jack, MD (Principal Investigator), Ann Ammons, BS, John Crisler, Chimaroke Edeoga, MBBS, MPH, Sotonte Ebenibo, MBBS, MPH, Ebenezer Nyenwe, MBBS, Jim Wan, PhD. Past members: Emmanuel Chapp-Jumbo, MBBS (2009–2011), Ruben Cuervo, MD (2006–2007), Nonso Egbuonu, MBBS (2007–2010), Nicoleta Ionica, MD (2007–2008), Dorota Malinowski, MD (2007–2008), Gabrielle Songe, BA (2008–2010). Consultant: Steven Haffner, MD; Data and Safety Officer: Murray Heimberg, MD, PhD.
